# Association of the Weight-Adjusted-Waist Index With Risk of All-Cause Mortality: A 10-Year Follow-Up Study

**DOI:** 10.3389/fnut.2022.894686

**Published:** 2022-05-25

**Authors:** Shuang Cai, Lin Zhou, Yue Zhang, Bokai Cheng, Anhang Zhang, Jin Sun, Man Li, Yongkang Su, Qiligeer Bao, Yan Zhang, Shouyuan Ma, Ping Zhu, Shuxia Wang

**Affiliations:** ^1^Department of Geriatrics, the 2nd Medical Center, Chinese PLA General Hospital, Beijing, China; ^2^Medical School of Chinese PLAl, Beijing, China; ^3^Department of Outpatient, the First Medical Center, Chinese PLA General Hospital, Beijing, China

**Keywords:** weight-adjusted waist index, all-cause mortality, obesity, older people, 10-year follow-up

## Abstract

**Background:**

To explore the relationship between weight-adjusted-waist index (WWI) and the risk of all-cause mortality in one urban community-dwelling population in China.

**Methods:**

This is a prospective cohort study with a sample of 1,863 older adults aged 60 years or over in Beijing who completed baseline examinations in 2009–2010 and a 10-year follow-up in 2020. WWI was calculated as waist circumference (cm) divided by the square root of weight (kg). Cox regression analysis was performed to investigate the significance of the association of WWI with all-cause mortality. The area under the receiver operating characteristic (ROC) curves were used to compare the ability of each obesity index to predict mortality.

**Results:**

During a median follow-up of 10.8 years (1.0 to 11.3 years), 339 deaths occurred. After adjusted for covariates, the hazard ratios (HRs) for all-cause mortality progressively increased across the tertile of WWI. Compared with the lowest WWI category (tertile1 <10.68 cm/√kg), with WWI 10.68 to 11.24cm/√kg, and≥11.25 cm/√kg, the HRs (95% confidence intervals (CIs)) for all-cause mortality were 1.58 (1.12–2.22), and 2.66 (1.80–3.92), respectively. In stratified analyses, the relationship between WWI and the risk of all-cause mortality persisted. The area under ROC for WWI was higher for all-cause mortality than BMI, WHtR, and WC.

**Conclusion:**

WWI was associated with a higher risk for all-cause mortality, and the association was more robust with the highest WWI category.

## Introduction

Globally, obesity is a significant health challenge as it greatly increases the risk of developing diseases such as metabolic disease, hypertension, stroke, myocardial infarction, osteoarthritis, Alzheimer's disease, depression, and several cancers, resulting in a reduction in life expectancy, with an estimated loss of 5–20 years (1–3). The incidence of obesity is on the rise worldwide. The global prevalence of obesity has almost tripled since the 1980s and continues to grow rapidly (4–6).

Body mass index (BMI) is a widely used measure of obesity. While BMI measures an individual's health, it is flawed. Its assessment of body fat in older adults is not as accurate as of that in younger adults. It doesn't distinguish visceral fat from fat in other areas, such as the buttocks (7). The accumulation of visceral fat, rather than subcutaneous fat, has been strongly associated with insulin resistance, high blood pressure, and dyslipidemia; excess visceral fat is more harmful to an individual's health than fat elsewhere (8–10). Much evidence indicated a linear association between BMI and the risk of hypertension, type 2 diabetes, and cardiovascular diseases (CVD) (11). However, inconsistent or inverse associations between BMI and mortality in various populations have contributed to the “obesity paradox” (12–14). Waist circumference (WC) has been proposed as a high association with CVD risk factors and mortality (15, 16). However, the “obesity paradox” was also found when WC was used to measure obesity (17). Moreover, WC is highly correlated with BMI and is thus limited as an independent measure of mortality risk (16, 18, 19). Waist-to-Height Ratio (WHtR) appears superior in assessing obesity but remains controversial in predicting obesity-related CVD risk and mortality (20–22).

A new adiposity index termed the “weight-adjusted-waist index” was proposed in this context (23). WWI has been proposed to assess obesity (24). Studies on about 1 million Korean adults found that WWI was positively correlated with CVD mortality; unlike BMI, WC, and waist-to-hip ratio, WWI was best at predicting the risk of cardiometabolic disease and death (23). However, the relationship between WWI and all-cause mortality in older Chinese is unclear. Therefore, this study aimed to investigate the association between WWI and all-cause mortality in elderly Chinese.

## Methods

### Study Population

Our research program and sampling details have been described above (25–27). In brief, this community-based study was conducted in the Wanshou Road Community of Haidian District in Beijing from September 2009 to June 2010. A two-stage stratified cluster sampling selected community residents aged 60 or above as a representative sample. We excluded cancer patients at baseline. Most cancers are chronic wasting diseases, resulting in weight loss and a reduction in WC (28, 29), there will be an impact on the primary study indicators. A total of 2,162 subjects (female:60.1%) completed the survey. Of the study population, 19 subjects were excluded due to lack of anthropometric data, 280 were lost during the follow-up period from 2010 to 2020, and 1,863 were finally available for statistical analysis. As previously mentioned, all participants in this study were recruited in a community-based cross-sectional survey. Therefore, we retrospectively divided the subjects into three groups to calculate the test power, applied sample size, hazard ratio, overall probability of event, proportion of sample in group, power value was 0.85 and 0.99 and concluded that the difference was statistically significant.

The study protocol was reviewed and approved by the ethical committees of the Chinese PLA General Hospital. The research procedures followed the ethically normative criteria. Written informed consent was acquired from all subjects. All investigators were trained at the Chinese PLA General Hospital and qualified for the post.

### Outcome Measures

The outcomes in the present study were all-cause mortality. All-cause death is defined as death from any cause. Follow-up ended in December 2020, and survival was defined as the number of months from recruitment to death or the end of observation (December 31, 2020). Information about vital status was determined through telephone interviews with family members or other caregivers. Respondents' identities were verified by information such as name, age, and gender.

### Data Collection

Using standard questionnaires, the researchers assessed the demographics of all participants through face-to-face interviews, including a range of demographic factors, medical history, and lifestyle. Lifestyle includes drinking and smoking. Alcohol consumption and smoking were considered dichotomous variables for never/former and current. Height, weight, WC, and blood pressure were measured according to standardized protocols. Anthropometric measurements were taken by specially trained researchers on subjects wearing light clothes and no shoes. Weight and height were measured twice to the nearest 0.1 kg and 0.1 cm, respectively. We measured the WC of the standing subjects with a piece of soft tape located between the lowest rib and the iliac crest (to the nearest 0.1 cm). WWI was calculated as WC (cm) divided by the square root of weight (kg) (23). BMI was calculated as weight in kilograms divided by the square of height in meters.

Two blood pressure recordings (5-min intervals) were obtained from participants' right arms in a sitting position after 30 min of rest. The blood pressure was measured using a sphygmomanometer, and the average of the two was used for analysis.

Fasting blood samples were taken from all subjects in the morning (after fasting for at least 12 h). An automatic biochemical analyzer measured serum lipids, glucose, routine blood tests, and creatinine. All biochemical analyses were performed in the Department of Biochemistry of the Chinese People's Liberation Army General Hospital.

Participants with fasting plasma glucose ≥7.0 mmol/l or 2-h plasma glucose ≥11.1 mmol/l after oral glucose tolerance test or blood glucose ≥11.1 mmol/L at any time or those receiving anti-diabetic medications were diagnosed with diabetes mellitus (30, 31). The study population included patients with type 2 diabetes.

### Statistical Analysis

Statistical Package for the Social Sciences (SPSS) software (version 26.0) was used for data management and statistical analysis. Continuous variables were reported as mean ± standard deviation (SD) and categorical variables as percentages. WWI (cm/√kg) was classified by tertile as follows: tertile 1 (<10.68 cm/√kg), tertile 2 (10.68–11.24 cm/√kg), and tertile 3 (≥11.25 cm/√kg). Baseline characteristics between subjects in different groups were compared using the χ2 test and analysis of variance. We investigated the all-cause mortality according to the WWI categories, with the lowest WWI category (<10.68 cm/√kg) as the reference. Cox proportional hazards models were used to estimate the association between all-cause mortality risk and WWI, estimating HRs and 95% CIs. We developed three models to adjust for potential confounders and plotted survival curves. Model 1: unadjusted; Model 2: adjusted for sex, age; Model 3: adjusted for age, sex, BMI, WC, systolic blood pressure (SBP), diastolic blood pressure (DBP), fasting plasma glucose (FPG), total cholesterol (TC), high-density lipoprotein (HDL-C), low-density lipoprotein cholesterol (LDL-C), triglycerides (TG), serum uric acid (SUA), Serum creatinine (Scr), smoking, alcohol drinking, coronary heart disease (CHD), hypertension (HTN), diabetes, stroke, Subgroup analyses were stratified by sex (men or women), age (<75 or ≥75 years), smoking status (smoking or no smoking), drinking status (alcohol drinking or no alcohol drinking), BMI (<24 kg/m^2^ or 24 kg/m^2^). Findings were recorded by HRs and 95% CIs. The area under the ROC curves were used to compare the ability of obesity indexes to predict mortality. All statistical tests were 2-sided, with *P* < 0.05 considered statistically significant.

## Results

### Baseline Characteristics

Among 1,863 participants (40.69% males), the median follow-up time was 10.8 years (1.0 to 11.3 years), and 339 deaths occurred. The cumulative incidence of death during follow-up was 18.2%. Table 1 shows the baseline characteristics of participants by WWI categories: As compared with the lowest WWI category (<10.68 cm/√kg), the highest WWI category (≥11.25 cm/√kg) had higher BMI, WC, SBP, DBP, FPG, TG, SUA, but lower HDL-C; were more likely to be older and probably had a higher rate of diabetes, HTN, and stroke (all *P* < 0.05).

**Table 1:** Baseline characteristics of study participants by weight-adjusted waist index.

	**Weight-adjusted waist index(cm/√kg)**			
**Characteristics**	**Tertile1(<10.68)**	**Tertile2(10.68-11.24)**	**Tertile3(≥11.25)**	***P-*value**
*N*	621	617	625	
Age (years)	68.56 ± 6.78	70.68 ± 6.70	73.81 ± 6.61	<0.001
Males (%)	288(46.4)	278(45.1)	192(30.7)	<0.001
BMI (kg/m2)	24.20 ± 3.11	25.11 ± 3.38	25.58 ± 3.64	<0.001
WC (cm)	81.96 ± 8.48	88.69 ± 7.44	93.49 ± 8.45	<0.001
SBP (mmHg)	135.05 ± 17.04	138.51 ± 19.44	141.18 ± 20.77	<0.001
DBP (mmHg)	76.51 ± 9.20	77.57 ± 9.98	77.33 ± 9.96	0.132
FPG (mmol/L)	5.83 ± 1.23	6.01 ± 1.45	6.26 ± 1.84	<0.001
TC (mmol/L)	5.22 ± 0.99	5.23 ± 1.02	5.28 ± 1.01	0.544
TG (mmol/L)	1.53 ± 0.90	1.66 ± 0.80	1.76 ± 0.98	0.005
HDL-C (mmol/L)	1.46 ± 0.40	1.39 ± 0.35	1.40 ± 0.40	0.007
LDL-C (mmol/L)	3.23 ± 0.84	3.24 ± 0.86	3.21 ± 0.87	0.896
SCr (μmol/L)	74.53 ± 19.13	74.01 ± 20.43	74.39 ± 22.16	0.911
SUA(μmol/L)	300.34 ± 79.68	308.79 ± 92,78	318.33 ± 91.74	0.002
Smokers (%)	183(29.5)	201(32.6)	180(28.8)	0.300
Drinkers (%)	176(28.3)	153(24.8)	140(22.4)	0.052
CHD, *n* (%)	130(20.9)	146(23.7)	160(25.6)	0.148
HTN, *n* (%)	299(48.1)	349(56.6)	368(58.9)	<0.001
Diabetes, *n* (%)	86(13.8)	113(18.3)	143(22.9)	<0.001
Stroke, *n* (%)	61(9.8)	82(13.3)	89(14.2)	0.045

*BMI, body mass index; WC, waist circumference; SBP, systolic Blood pressure; DBP, diastolic blood pressure; FPG, fasting plasma glucose; TC, total cholesterol; HDL-C, high-density lipoprotein; LDL-C, low-density lipoprotein cholesterol; TG, Triglycerides; SUA, serum uric acid; Scr, Serum creatinine; CHD, Coronary heart disease; HTN, hypertension*.

### Association Between WWI and All-Cause Mortality

**Figure 1:**
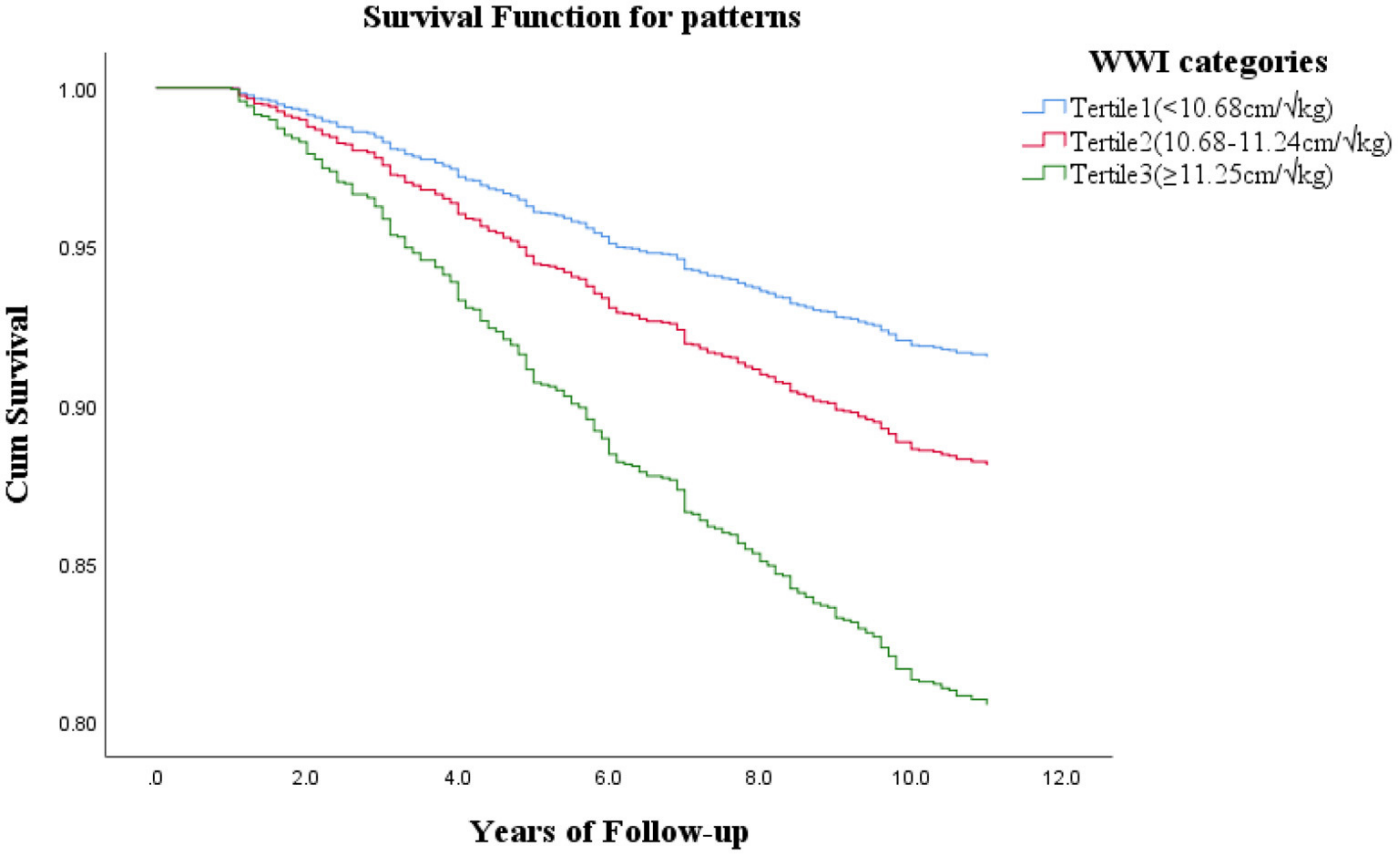
Cox proportional hazards after adjusted for age, sex, BMI, WC, SBP, DBP, FPG, TC, HDL, LDL, TG, SUA, SCr, smoking, alcohol drinking, CHD, HTN, diabetes, stroke.

Table 2 and Figure 1 show the association between WWI and the risk of all-cause mortality. For all models, the WWI category was positively associated with all-cause mortality. Univariate analysis (model 1), after adjustment for age and sex (model 2), and further for BMI, WC, SBP, DBP, FPG, TC, HDL, LDL, TG, SUA, Scr, smoking, alcohol drinking, CHD, HTN, diabetes, stroke (model 3), the HRs for all-cause mortality remained progressively increased across tertile of WWI. Specifically, as compared with the lowest WWI category (<10.68 cm/√kg), a WWI of 10.68 to 11.24 cm/√kg increased the probability of all-cause mortality (HR 1.58, 95% CI 1.12–2.22), as did the highest WWI category (≥11.25 cm/√kg) (HR 2.66, 95% CI 1.80–3.92).

**Table 2:** Cox regression analyses for the association between all-cause mortality and weight-adjusted waist index.

	**HR (95% CI)**						
**WWI (cm/√kg)**	**Cases/participants**	**Model 1**	***P*-value**	**Model 2**	***P*-value**	**Model 3**	***P*-value**
Tertile1(<10.68)	66/621	1 (References)		1 (References)		1 (References)	
Tertile2(10.68–11.24)	108/617	1.71(1.26–2.32)	0.001	1.51(1.11–2.05)	0.009	1.58(1.12–2.22)	0.009
Tertile3(≥11.25)	165/625	2.74(2.06–3.65)	<0.001	2.45(1.83–3.29)	<0.001	2.66(1.80–3.92)	<0.001

*Values are n or HR (95% CI)*.

*HR, hazard ratio; CI, confidence interval*.

*Model 1: Unadjusted*.

*Model 2: Adjusted for sex, age*.

*Model 3: Adjusted for age, sex, BMI, WC, SBP, DBP, FPG, TC, HDL, LDL, TG, SUA, SCr, smoking, alcohol drinking, CHD, HTN, diabetes, stroke*.

### Subgroup Analyses for Association Between WWI and All-Cause Mortality

**Figure 2:**
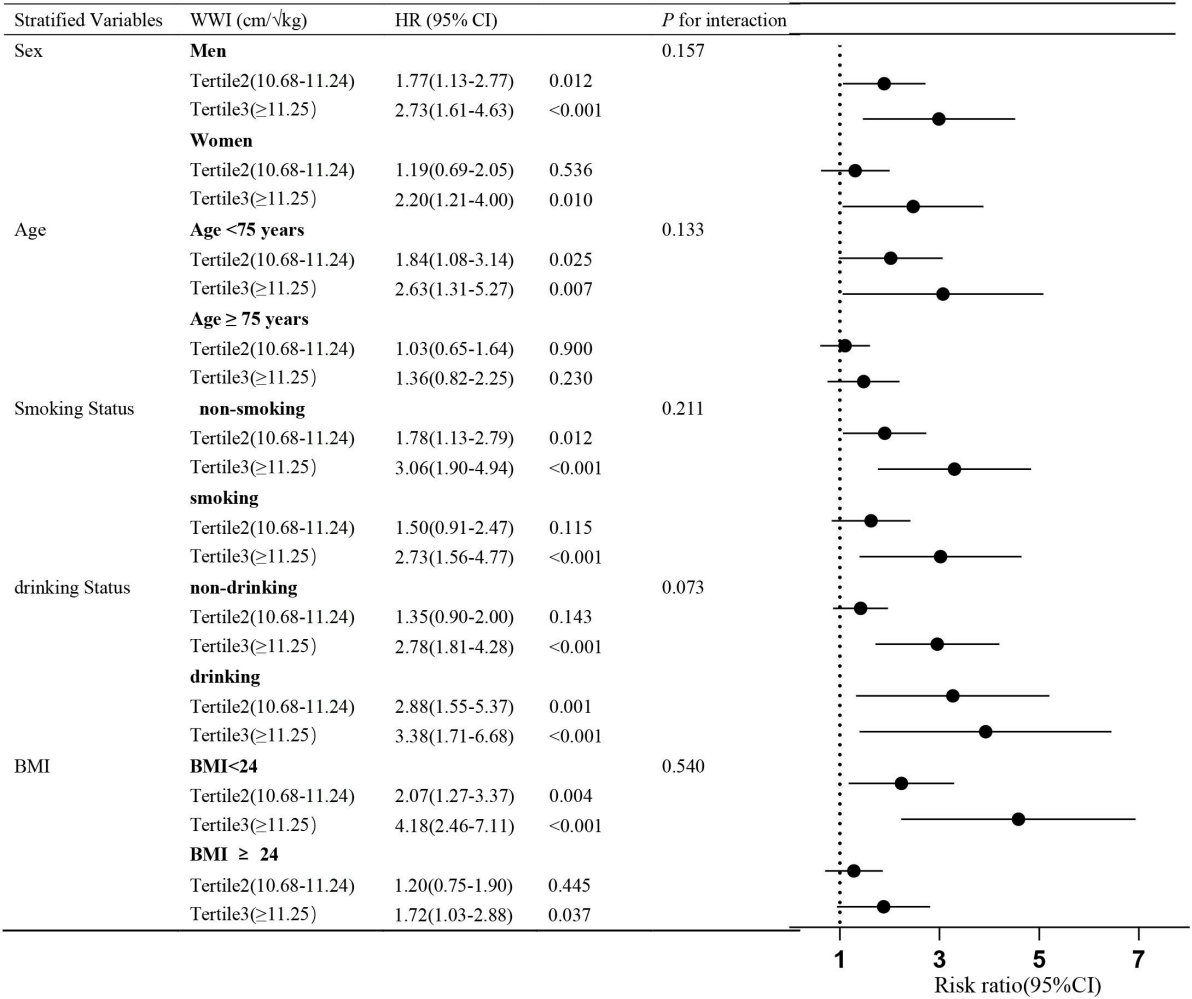
Subgroup analyses for the association between all-cause mortality and WWI categories. Adjusted for age, sex, BMI, WC, SBP, DBP, FPG, TC, HDL, LDL, TG, SUA, smoking, alcohol drinking, CHD, HTN, diabetes, stroke, except for the stratified variable. The stratified analysis was performed according to the boundaries of the bolded values.

The association between the highest WWI category (≥11.25 cm/√kg) and the risk of all-cause mortality was evaluated by the subgroups sex, age, smoking status, drinking status, BMI, with the lowest WWI category (<10.68 cm/√kg) as the reference. After controlling for all covariates except for the stratified variable, the association between WWI and the risk of all-cause mortality remained in nearly every subgroup analysis, but the association was not significant at age ≥75 years (Figure 2).

### ROC Curves of Each Obesity Indices

**Figure 3:**
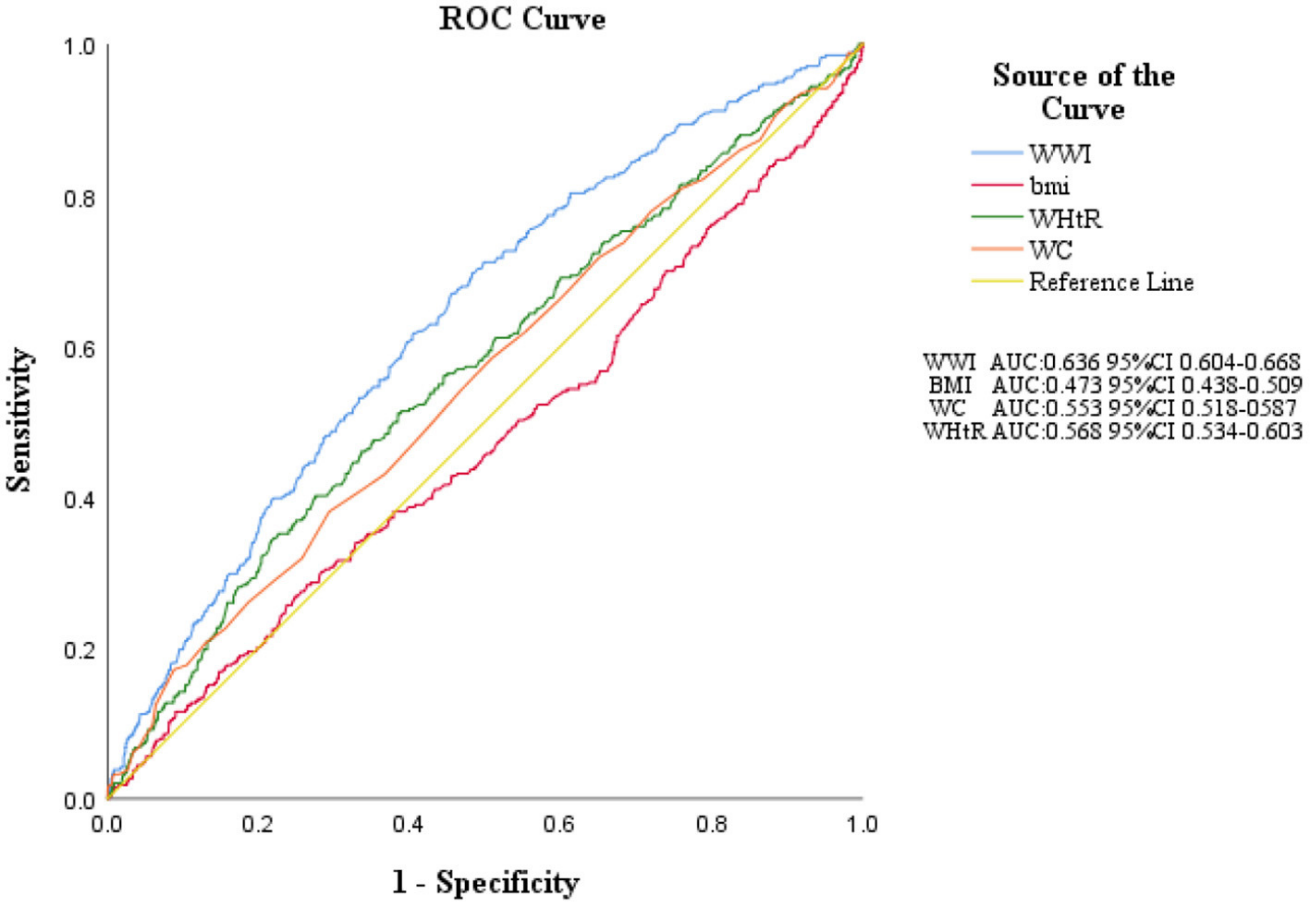
ROC curves of each obesity indices. WWI, weight-adjusted-waist index; BMI, body mass index; WHtR, waist-to-height ratio; WC, waist circumference; ROC, receiver operating characteristic; AUC, area under the curve.

Figure 3 shows the area under the ROC curve with its 95% CI to identify all-cause mortality by each obesity indices. According to the ROC analyses, WWI AUC value: 0.636, WC: 0.553, WHtR:0.568. However, the AUC of BMI had no statistical significance for all-cause mortality (AUC <0.5).

## Discussion

In this large prospective study of an older Chinese population with a 10-year follow-up cohort, we suggested that WWI, a new adiposity index, was significantly associated with an increased risk of all-cause mortality. The association was independent of sex, age, lifestyle factors, BMI, WC, and various cardiovascular and cerebrovascular risk factors. The highest WWI category (≥11.25 cm/√kg) was 2.66-fold associated with all-cause mortality as compared with the lowest WWI category (<10.68 cm/√kg). These results were essentially consistent in subgroup analyses. The association between WWI and all-cause mortality in older people (≥75years) disappeared. This result may be due to differences in body fat distribution between the older and the younger. In addition, there are more risk factors for death in the elderly population. Furthermore, this inconsistent result may also be due to the limited statistical power of this study. Finally, our findings showed that WWI was superior to BMI, WC, and WHtR in predicting all-cause mortality.

Obesity is caused by a chronic energy imbalance between eating too many calories and burning too few (32). Other studies have suggested that obesity may be an inherited disorder of energy homeostasis (33). Hormonal, nutritional, and metabolic factors, energy expenditure, psychological factors, and sedentary behavior play a role in the pathophysiology of obesity (34). Obesity prevalence increases year by year and has become the number one lifestyle-related risk factor for premature death (5). Several studies have demonstrated that obesity increases the risk of all-cause mortality (35–39).

In 1993, WHO defined obesity as a BMI≥30 kg/m^2^ (40). However, subsequent evidence suggests that Asians with lower BMIs are more likely to develop diabetes than European-American (41). Well-known, death rates are highest in those who have had diabetes for a long time (42). A recent large study found that the risk of diabetes in Chinese people with a BMI of 26.9 kg/m^2^was the same as that of European-American populations with a BMI of 30 kg/m^2^, which remained after controlling for socioeconomic status and smoking status (41, 43). It is unclear whether the lower threshold for BMI in Asians is due to differences in body composition, biochemical characteristics, lifestyle, or genetics. However, what is clear is that Asian populations need to intervene with weight change earlier. Unfortunately, current mainstream guidelines do not make clear racial distinctions, which ignores east-west differences and other factors such as economics. In addition, whether BMI is the best predictor of future health is debatable, so more detailed studies are urgently needed to guide clinical practice.

WWI is a new obesity index based on weight and WC, which has a good predictive ability for cardiometabolic morbidity and mortality in the Korean population (23). Li et al. recently reported in a rural Chinese cohort study that the highest WWI category was significantly associated with an increased risk of HTN (44). A recent study on WWI reflecting fat and muscle mass in the opposite direction in older adults showed that WWI was positively correlated with fat mass and negatively correlated with muscle mass (24). WWI is a novel obesity index developed in recent years, and there are few related studies. Our study further verified the association of this indicator with all-cause mortality in the Chinese elderly population. The indicator is easy to operate and economical. It can be applied to medical and health institutions at all levels, especially in areas where medical standards are lacking or large-scale data research is required. Therefore, for the high WWI population, early assessment of target organ damage and initiation of treatment can reduce the risk of cardiovascular and cerebrovascular diseases and improve prognosis.

The present study has some limitations. Firstly, our sample did not include young adults, and more research is needed to validate our results for the general population. Secondly, confounding variables not included in the current analyses, such as socioeconomic status and some drugs possibly affecting mortality. All individuals were chosen from the Wanshoulu Community, and their socioeconomic status is relatively balanced. However, some drugs were not included in the study due to their wide variety and low usage rate. Finally, the association between WWI and cause-specific mortality could not be determined because death registration details were unavailable.

## Conclusion

In this study, high levels of WWI were positively associated with all-cause mortality in older Chinese people. This study may provide evidence that WWI as an indicator of obesity is an independent risk factor for all-cause mortality, suggesting that WWI may be an intervention indicator to reduce all-cause mortality in the elderly.

## Data Availability Statement

The original contributions presented in the study are included in the article/supplementary materials, further inquiries can be directed to the corresponding author/s.

## Ethics Statement

The studies involving human participants and was performed in accordance with the Declaration of Helsinki and was approved by the Medical Ethics Committee of Chinese PLA General Hospital. Informed written consent to participate in the study was obtained from all participants. The patients/participants provided their written informed consent to participate in this study.

## Author Contributions

SW, PZ, and SC designed the research. SC, BC, and AZ collected the data. SC, LZ, and YuZ wrote the manuscript. JS, ML, YS, YaZ, SM, and QB help optimize the research and proofread the manuscript. All authors read and approved the final manuscript.

## Funding

This study was supported by the “National Key R&D Program of China” (Funding No. 2020YFC2008900), the Military Medical Youth Growth Project of PLA General Hospital (Funding No. QNC19005), which contributed to the data collection job.

## Conflict of Interest

The authors declare that the research was conducted in the absence of any commercial or financial relationships that could be construed as a potential conflict of interest.

## Publisher's Note

All claims expressed in this article are solely those of the authors and do not necessarily represent those of their affiliated organizations, or those of the publisher, the editors and the reviewers. Any product that may be evaluated in this article, or claim that may be made by its manufacturer, is not guaranteed or endorsed by the publisher.

## Abbreviations

WWI: weight-adjusted-waist index
ROC: receiver operating characteristic
BMI: body mass index
WHO: World Health Organization
CVD: cardiovascular diseases
WC: waist circumference
WHtR: Waist-to-Height Ratio
SBP: systolic Blood pressure
DBP: diastolic blood pressure
FPG: fasting plasma glucose
TC: total cholesterol
HDL-C: high-density lipoprotein
LDL-C: low-density lipoprotein cholesterol
TG: Triglycerides
SUA: serum uric acid
Scr: Serum creatinine
CHD: Coronary heart disease
HTN: hypertension
SD: Standard deviation
HRs: hazard ratios
95%CIs: 95% confidence intervals.
